# A Search for Mitochondrial Damage in Alzheimer’s Disease Using Isolated Rat Brain Mitochondria

**Published:** 2016

**Authors:** Mehrdad Faizi, Enayatollah Seydi, Sadegh Abarghuyi, Ahmad Salimi, Sanaz Nasoohi, Jalal Pourahmad

**Affiliations:** Department of Pharmacology and Toxicology, Faculty of Pharmacy, Shahid Beheshti University of Medical Sciences, Tehran, Iran.

**Keywords:** Alzheimer disease, amyloid-β peptide, Mitochondria, Oxidative stress, Apoptosis

## Abstract

Alzheimer’s disease (AD) is a progressive neurodegenerative disorder that affects regions of the brain that control cognition, memory, language, speech and awareness to one’s physical surroundings. The pathological initiation and progression of AD is highly complex and its prevalence is on the rise. In his study, Alzheimer's disease was induced with single injection of amyloid-β (Aβ) peptides (30ng, by stereotaxy) in each hemisphere of the Wistar rat brain. Then memory dysfunction, oxidative stress and apoptosis induced by Aβ peptide were investigated on isolated brain mitochondria obtained from infected rat. Our results showed memory impairment in rats after receiving an Aβ peptide. We also found significant rise (P<0.05) at ROS formation, mitochondrial membrane depolarization, mitochondria swelling, cytochrome c release and significant decrease in ATP/ADP ratio on mitochondria isolated from brain of these memory impaired rats compared with those of untreated control rat group. Activation of caspase-3 the final mediator of apoptosis in the brain homogenate of the memory impaired rats was another justification for occurrence of neuron loss in the experimental model of AD. Our results suggest that oxidative stress and mitochondria mediated apoptosis in brain neurons play very important role in initiation of AD.

## Introduction

 Alzheimer’s disease (AD) is a progressive and irreversible neurodegenerative disease described by the presence of two abnormal structures in the brain of the patients, amyloid-β (Aβ) plaques and tau neurofibrillary tangles (1-2). AD is main cause of dementia in the middle-aged and elderly (3). About 10% of the population older than 65 years of age show AD syndromes (4), and it is an important healthcare concern (2). Also this disease characterized by progressive memory dysfunction and decreased cognitive performance (5). In addition to the medical severity of AD, the annual cost the disease care worldwide exceeds $600 billion. At this time, more than 36 million people suffering from dementia, and this number is expected to triple by 2050 (3). In spite of study in animal models of AD, the disease remains incompletely understood, with few treatment choices (3). 

Although cellular and molecular pathology and etiology of AD have not yet been completely understood, previous studies reported that mitochondrial dysfunction and oxidative stress may play roles in the development of the disease (6-7). Many studies have suggested that mitochondrial abnormalities such as increased oxygen species generation and deficient mitochondrial dynamic balance are an event in AD (1). One study reported that mutations in mitochondrial DNA (mtDNA) as the basis for AD (8). Mitochondria are responsible for more than 90% of the cellular ATP generation (9). This bioenergetics assumes its maximum significance in the brain and is essential for neuronal function (6, 9). Mitochondria are involved in vital cellular functions that are important for survival and death (9).

Animal models offer valuable tools for evaluating new therapeutic strategies for the treatment of human diseases, as well as for the studying of pathological mechanisms involved in the disease processes. Behavioral scientists favor the rat because it is an intelligent and quick learner experimental animal. Direct injection of Aβ peptide into the rat brain is an experimental model for AD induction (10). Behavioral tests such as Morris water maze, Y‑maze and radial arm maze are used to assess memory functions in AD models (11). To understand the role of mitochondrial function in AD, it is necessary to generate human cellular models which involve living neurons (1).

In this study we used Aβ peptide intra brain injection for induction of AD in wistar rat, and then we isolated mitochondria from brain of AD rats to figure out probable mitochondrial toxic mechanisms involved in the neuronal pathology of AD such as increased active oxygen radicals (ROS) formation, collapse of mitochondrial membrane potential and ADP/ATP ratio, mitochondrial swelling, release of cytochrome c and finally cell death signaling by caspase-3 activation. 

## Experimental

*Animals*

Male wistar rats (250-300 g) were housed in an air-conditioned room with controlled temperature of 25 ± 2◦C and maintained on a 12:12 h light cycle with free access to food and water. All experimental procedures were conducted according to the ethical standards and protocols approved by the Committee of Animal Experimentation of Shahid Beheshti University of Medical Sciences, Tehran, Iran. All efforts were made to minimize the number of animals and their suffering.

*Experimental design*

Male wistar rats were divided into two groups, comprising 10 animals each, as follows: group A includes untreated control rats, Group B (disease model) include amyloid-β (Aβ) peptide treated rats whom were injected with single dose of amyloid-β (Aβ) peptide (30 ng, by stereotaxic method) in each hemisphere of the brain. 

**Figure 1:**
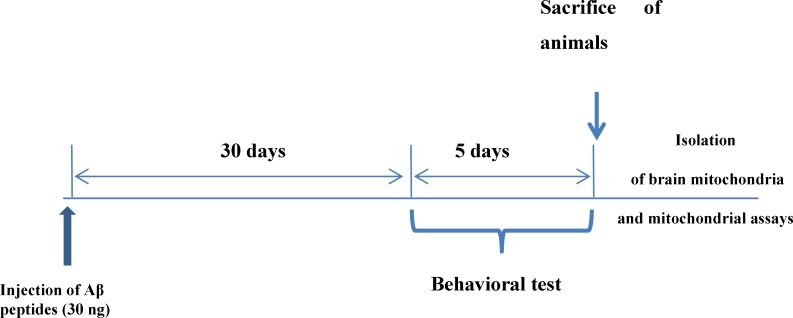
Experimental protocol.

*Behavioral test*

*Morris water maze*

30 days after injection of Aβ peptides, behavioral testing was performed. This test measures spatial reference memory. In this test a mouse or a rat is placed into a circular tank containing water, and has to find an escape platform in a fixed place just beneath the surface. The escape platform is not visible to the animal because the water has been rendered opaque. After swimming around for a certain time, the animal will eventually come across the hidden platform and climb onto it to escape from the water. When placed again in the water on subsequent occasions, the animal will generally find the platform with increasing rapidity, indicating that it has learned the position of the platform. There are different ways to perform the test and also many parameters to assess memory, including path length and time to find the platform (11-12).

*Mitochondrial preparation*

After significant appearance of behavioral dysfunction by probe trial test in Aβ peptides treated test group rats, all animals in both test and control groups were sacrificed by cervical decapitation. Then, mitochondria were prepared from wistar rat’s brain using differential centrifugation (13-14). The brain was removed and minced with small scissors in a cold mannitol (225 mM) solution (mitochondria isolation buffer). The minced brain was gently homogenized in a glass homogenizer with a Teflon pestle and then centrifuged at 1500 × g for 10 min at 4 °C for the removal of nuclei, unbroken cells, and other non-subcellular tissues and the pellet was discarded. The supernatant was subjected to a further centrifugation at 12,000×g for 10 min and the superior layer was carefully discarded. The mitochondrial pellet was washed by gently suspending in the isolation medium (0.225 M D-mannitol, 75 mM sucrose, and 0.2 mM EDTA, pH=7.4) and centrifuged again at 12,000×g for 10 min. Final mitochondrial pellets were suspended in Tris buffer containing (0.05 M Tris-HCl, 0.25 M sucrose, 20 Mm KCl, 2.0 mM MgCl_2_, and 1.0 mM Na_2_HPO_4_, pH of 7.4) at 4 °C, except for the mitochondria used to assess ROS production, MMP and swelling, which were suspended in respiration buffer (0.32 mM sucrose,10 mM Tris, 20 mM Mops, 50 μM EGTA, 0.5 mM MgCl_2_, 0.1 mM KH_2_PO_4_ and 5 mM sodium succinate), MMP assay buffer (220 mM sucrose, 68 mM D-mannitol, 10 mM KCl,5 mM KH_2_PO_4_, 2 mM MgCl_2_, 50 μM EGTA, 5 mM sodium succinate, 10 mM HEPES, 2 μM Rotenone) and swelling buffer (70 mM sucrose, 230 mM mannitol, 3 mM HEPES, 2 mM tris-phosphate, 5 mM succinate and 1 μM of rotenone). In our study the brains of each rat was homogenized and studied separately. 

Mitochondria were prepared fresh for each experiment and used within 4 h of isolation and all steps were strictly operated on ice to guarantee the isolation of high-quality mitochondrial preparation.

*Protein Concentration*

Mitochondrial protein concentration was determined by the Coomassie blue protein binding method (Bradford, 1976) using BSA as the standard (15). For the normalization process in all the following mitochondrial assays, the mitochondrial samples (0.5 mg mitochondrial protein/ml) were used.

*Determination of complex II activity through the MTT assay*

The activity of mitochondrial complex II (succinate dehydrogenase) was assayed through the measurement of MTT reduction and the absorbance at 570 nm was measured with an ELISA reader (Tecan, Rainbow Thermo, Austria) (16).

*Determination of mitochondrial ROS level*

The mitochondrial ROS measurement was performed using the fluorescent probe DCFH. Briefly, isolated brain mitochondria were placed in respiration buffer. Following this step, DCFH was added (final concentration, 10 μM) to mitochondria and then incubated for 10 min. Then, the fluorescence intensity of DCF was measured using Shimadzu RF-5000U fluorescence spectrophotometer at the λEx = 488 nm and λEm = 527 nm (17).

*Determination of the MMP*

Mitochondrial uptake of the cationic fuorescent dye, rhodamine 123 (Rh123), has been used for the estimation of mitochondrial membrane potential (18). The mitochondrial fractions (0.5 mg protein/ml) were incubated with 10 µM of Rh123 in MMP assay buffer. Then, the fluorescence was monitored using Shimadzu RF-5000U fuorescence spectrophotometer at the λEx=490 nm and λEm=535 nm (19).

*Determination of mitochondrial swelling*

Determination of mitochondrial swelling after the isolated mitochondria (0.5 mg protein/mL) was estimated through changes in light scattering as monitored spectrophotometrically at 540 nm (30 °C). Firstly, isolated mitochondria were suspended in swelling buffer. In the next step, the absorbance was measured at 549 nm at 10 min time intervals with an ELISA reader (Tecan, Rainbow Thermo and Austria). A decrease in absorbance indicates an increase in mitochondrial swelling (13).

*Release of cytochrome c assay*

The concentration of cytochrome c was determined by using the Quantikine rat/mouse cytochrome c immunoassay kit provided by R&D Systems, Inc. (Minneapolis, MN). Briefly, a monoclonal antibody specific for rat/mouse cytochrome c was pre-coated onto the microplate. Seventy-five ml conjugate (containing monoclonal antibody specific for cytochrome c conjugated to horseradish peroxidase) and 50 ml standard and positive control were added to each well of the microplate. One microgram protein from each supernatant fraction was added to the sample wells. All the standards, controls, and samples were added to the microplate in duplicate. After 2 h incubation, substrate solution (100 mL) was added to each well and incubated for 30 min. After incubation, 100 mL of stop solution was added to each well, and the optical density of each well was determined by the aforementioned microplate spectrophotometer set at 450 nm.

*Determination of caspase-3 activity*

Caspase-3 activity was determined in cell lysate of hepatocytes from different treatments using “Sigma’s caspase-3 assay kit (CASP-3-C)” (20). In brief, this colorimetric assay is based on the hydrolysis of substrate peptide, Ac-DEVD-*p*NA, through caspase-3. The released moiety (*p*-nitroaniline) has a high absorbance at 405 nm. The concentration of the *p*-nitroaniline (μM) released from the substrate is calculated from the absorbance values at 405 nm or from a calibration curve prepared with defined *p*-nitro aniline solutions (21).

*Assay of ATP/ADP ratio*

The ATP/ADP ratio level was measured by luciferase enzyme as described by Tafreshi et al. (22). Bioluminescence intensity was measured using Sirius tube luminometer (Berthold Detection System, Germany). The method was based on the measurement of time to the disappearance of emission; even under conditions of visual process control, this method made it possible to determine ATP contents in the range of 10–150 μg. Currently, the mechanism of this reaction has been studied in sufficient detail. It is based on the oxidation of D-luciferin in the presence of ATP and oxygen catalyzed by firefly luciferase. The emission spectrum in the region of 470–700 nm is asymmetric with a maximum at 562 nm. The emission intensity is proportional to the concentration of ATP.

*Statistical analysis*

Results are presented as mean ± SD. Assays were performed in triplicate and the mean was used for statistical analysis. Statistical significance was determined using the student t-test or one-way ANOVA test, followed by the post-hoc Tukey test when appropriate. Statistical significance was set at P < 0.05.

## Results

*Probe trial test*

**Figure 2 (A, B).**
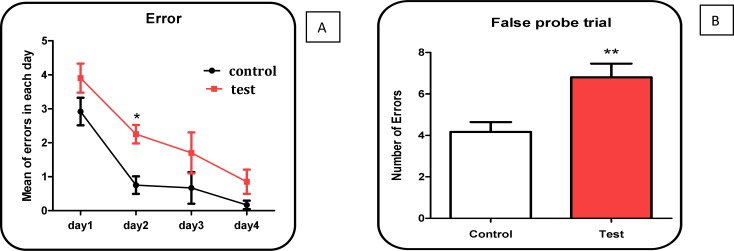
Number of errors in 4 days following treatment with Aβ peptide in treated rats compared with untreated control group. Values represented as mean ± SD (n = 3).^ *^ and ^**^ (P < 0.05 and P < 0.01, respectively); show significant difference in comparison with untreated control rat group

The results of our study by false probe test showed that mean of errors in Aβ peptide treated rat group was significantly (P < 0.05) higher than untreated control group (Figure 2 A and B).

Succinate dehydrogenase activity (complex II) assay

**Figure 3:**
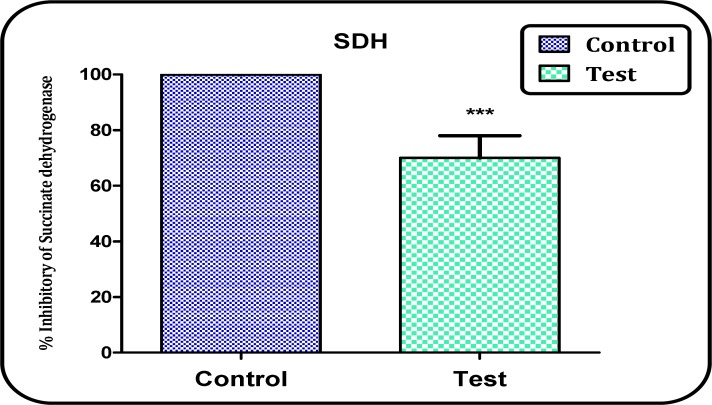
The Complex II (succinate dehydrogenase) activity in the brain mitochondria obtained from both Aβ peptide treated and untreated control rats. Values represented as mean ± SD (n = 3). ^*** ^P < 0.001 shows significant difference in comparison with control mitochondria

In this study, we determined the dehydrogenase activity by MTT test 1 h following isolation of mitochondria from brain of both Aβ peptide treated and control rats. Figure 3 shows a significant decrease in the mitochondrial metabolism of MTT to formazan in the brain mitochondria obtained from Aβ peptide treated rats compared to untreated control rats (P<0.05).

*Measurement of ROS*

**Figure 4:**
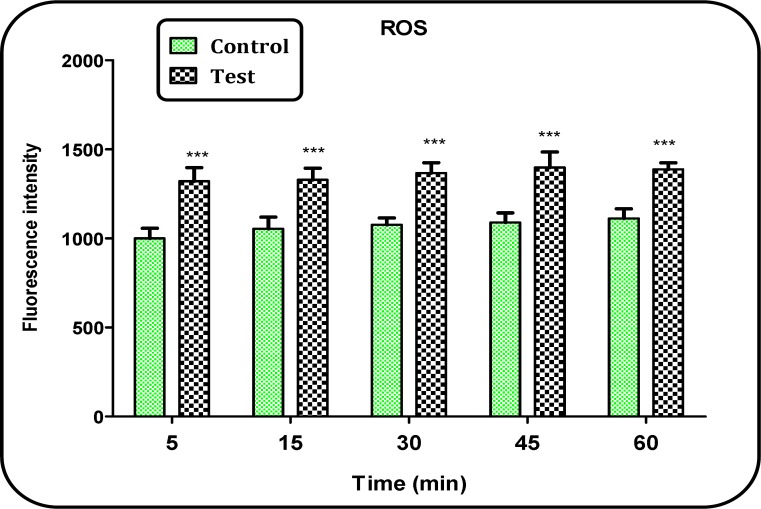
ROS formation in brain mitochondria isolated from both Aβ peptides treated and untreated control rats. ROS formation was measured fluorometrically using DCFH. Values are presented as mean ± SD (n = 3). ^***^ (P < 0.001); Significant difference in comparison with control mitochondria

As shown in Figure 4, ROS production was significantly (P < 0.05) increased in brain mitochondria isolated from Aβ peptide treated group compared to those of untreated control group. The ROS formation in both groups was measured in the time intervals (15, 30, 45 and 60 min) after isolation of brain mitochondria in both groups.

*Determination of Mitochondrial Membrane Potential (MMP)*

**Figure 5:**
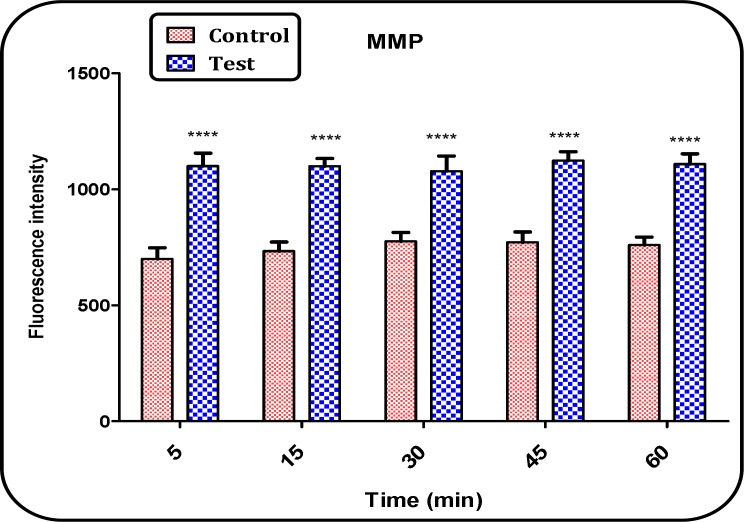
MMP decrease in the brain mitochondria isolated from both Aβ peptide treated and untreated control rat groups. MMP was measured by Rh 123 as described in Materials and methods. Values represented as mean ± SD (n = 3). ^**** ^(P < 0.0001) shows significant difference in compared with control mitochondria

MMP a highly sensitive indicator of the mitochondrial membrane depolarization was measured by Rh 123 redistribution. As shown in figure 4, MMP significantly (p < 0.05) decreased in the mitochondria isolated from brain of Aβ peptide treated group compared with those of untreated control group (Figure 5). The MMP in both groups was measured in the time intervals of 5, 15, 30, 45 and 60 min following the isolation of rat brain mitochondria.

*Measurement of Mitochondrial Swelling*

**Figure 6.**
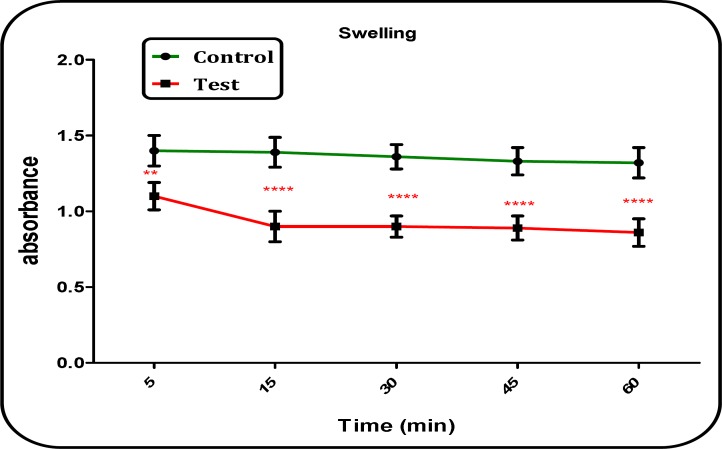
Mitochondrial swelling in the brain mitochondria isolated from both Aβ peptide treated and untreated control rat groups. Mitochondrial swelling was measured by determination of absorbance of mitochondrial suspension at 540 nm. Values represented as mean ± SD (n = 3). ^ ** ^and ^**** ^(P < 0.01 and P < 0.0001, respectively); significant difference compared with untreated control group mitochondria

Our results showed significant mitochondrial swelling (p < 0.05) in the mitochondria isolated from brain of Aβ peptide treated group compared with those of untreated control group (Figure 6). The mitochondrial swelling in both groups was measured in the time intervals of 5, 15, 30, 45 and 60 min following suspension of isolated rat brain mitochondria in the swelling buffer.

*Cytochrome c release*

**Figure 7:**
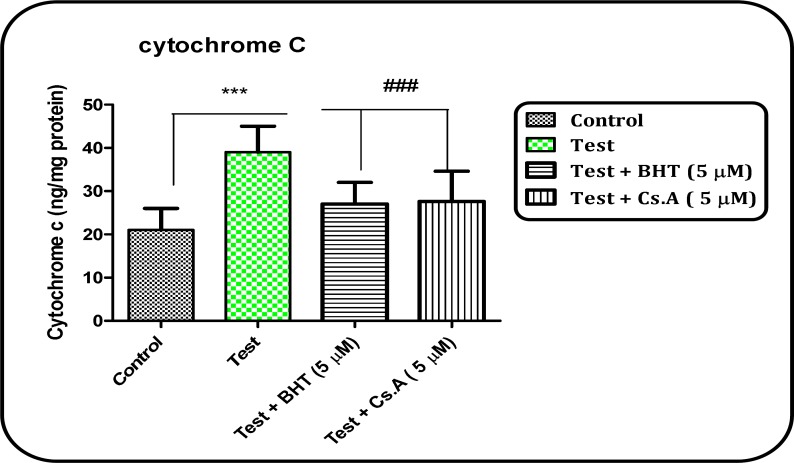
Cytochrome c release in the brain mitochondria isolated from both Aβ peptide treated and untreated control rat groups. Cytochrome c release was measured by ELISA kit as described in Materials and Methods. Values represented as mean ± SD (n = 3). ^*** ^(P < 0.001); significant difference compared with mitochondria isolated from untreated control rat group, and^ ###^ (P < 0.001); significant difference compared with mitochondria isolated from Aβ peptide treated rat group

As shown in Figure 7, that collapse of the mitochondrial membrane potential and disruption of mitochondrial outer membrane integrity significantly (P < 0.05) occurs in brain mitochondria isolated from Aβ peptide treated test group. The pretreatment of mitochondria isolated from Aβ peptide-treated rat group with an MPT inhibitor, Cs A or an antioxidant, BHT (Butylated hydroxytoluene) significantly (P < 0.05) inhibited cytochrome c release, indicating the role of oxidative stress and MPT pore opening in cytochrome c release.

*Caspase-3 activation*

**Figure 8:**
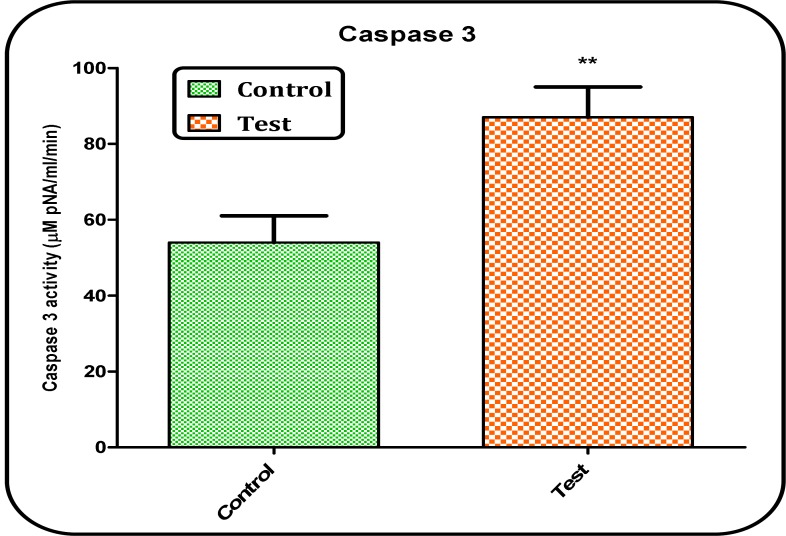
Determination of caspase-3 activity. Caspase-3 activity was determined by Sigma-Aldrich kit. The kit determines produced pNA that is released from the interaction of caspase-3 and AC-DEVD-pNA (peptide substrate). Values represented as mean ± SD (n = 3). ^** ^(P < 0.01); significant difference compared with untreated control rat group

The caspases are the most important effector molecules in the execution of apoptosis and progression of the caspases activation cascade ends in the activation of caspase-3, the final mediator of apoptosis. In this study, caspase-3 activity was significantly (p < 0.05) increased in brain tissue of Aβ peptides treated rat group (Figure 8).

*ATP/ADP ratio*

**Figure 9:**
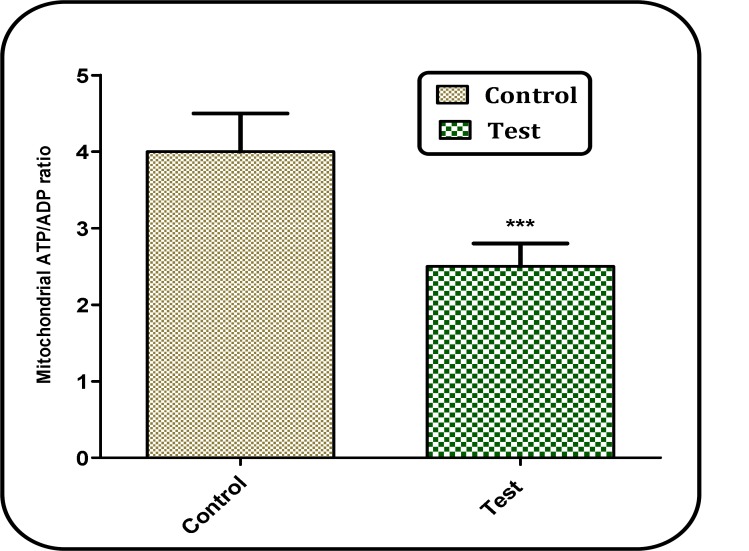
Determination of ATP/ADP ratio. ATP/ADP ratio was determined using Luciferin/Luciferase Enzyme System kit. Values represented as mean ± SD (n = 3). ^ *** ^(P < 0.001); significant difference compared with mitochondria obtained from untreated control rat group

In our study, we also measured the ATP/ADP ratios in brain mitochondria isolated from both Aβ peptide treated test and untreated control group. As shown in Figure 9, ATP/ADP ratio declined in brian mitochondria isolated from Aβ peptide treated rat group compared with those of untreated control rat group.

## Discussion

More than 35 million people are estimated to live with dementia worldwide. Between all dementing disorders, AD is the most prevalent, with estimated 24 million cases in the whole world and 5.5 million in the United States (23). AD is a disorder that often afflicts the elderly people of the world (24) and makes serious public health concern (25). This disease characterized clinically by deterioration of behavioral changes, cognitive functions, memory loss and motor dysfunction (23). The incidence of AD tends to increase in coming years as population’s age and life expectancy increases in both developed and developing countries (26). 

Some experimental models have so far been used to induce AD, such as intra brain injection of Aβ peptide. In this study, we used Aβ peptide for induction of AD in wistar rats. Our results showed that the number of errors in the Aβ peptides treated rats was significantly higher than that of untreated rats. The result (Morris water maze) of the present study seems to provide support for effect of Aβ peptide simulation AD in rats.

The mechanisms of neuronal degeneration in AD are not completely elucidated, and existing information is incomplete. In recent years, increasing consideration has been given to the role of mitochondrial dysfunction in the pathogenesis of different neurodegenerative disorders (27).There is much evidence of impaired mitochondrial function as a causative agent in neurodegenerative diseases (28). Several documents in the current literature show that the main elements of mitochondrial dynamics are changed in AD (29). One previous study reported that several mitochondrial enzymes (such as pyruvate dehydrogenase complex, ketoglutarate dehydrogenase complex, and cytochrome oxidase (CO)) are altered in AD (30). 

In this study we examined the activity of complex II (succinate dehydrogenase) in the mitochondria isolated from Aβ peptide treated group and compared it with that of untreated control group. Our result showed that activity of complex II (succinate dehydrogenase) significantly (P<0.05) reduced in the brain mitochondria isolated from Aβ peptide treated rat group.

Mitochondria play a central role in ROS production; which are derived from electrons leaking from the electron transport chain; a process closely connected with ATP production (27). Our findings by fluoremetric technique showed that brain mitochondrial ROS generation in the Aβ peptide treated group was significantly (P<0.05) higher than those of the untreated control group. This is also in agreement with another published work indicated that ROS could play a role in AD (6). Although basal ROS levels are essential for synaptic plasticity and memory, abnormally elevated ROS levels are in close connection with mitochondrial dysfunction, reported in AD neurons and in AD models (26). ROS are potent inducers of the mPTP formation and on the other hand, mPTP formation by itself raises ROS formation, decreases ATP production and releases apoptogenic compounds from mitochondria into cytosol accompanied by mitochondrial swelling (31). The results from this study confirmed all above referenced events. 

ROS and mitochondria play an upstream role in apoptosis induction. Interestingly, mitochondria are both source and target of ROS. Cytochrome c release from mitochondria, that triggers caspases activation, appears to be mainly mediated by ROS action (32). Our results showed that cytochrome c release in the mitochondria obtained from Aβ peptide treated group was significantly (P<0.05) higher than those of the untreated control group. Oxidation of the glutathione thiol groups surrounding mitochondrial MPT pores by ROS could contribute to cytochrome *c *release due to disruption of the MMP and MPT pores opening (32). Our results also provided evidence that mitochondrial ROS formation and apoptosis signaling are involved in cellular pathology of AD.

Apoptosis plays a main role during brain formation to eliminate the neurons that do not establish proper wiring in the developing neuronal network. It also occurs and could actually play an active role in the development of neurodegenerative diseases such as Parkinson’s diseases (33). Caspases are a family of genes important for regulatory homeostasis through controlling cell inflammation and death. Caspase-3 (a final executioner caspase) is an important component in some apoptosis pathways (34). Considering the fact that activation of caspases could be responsible for the neurodegeneration associated with AD, caspases are possible therapeutic targets for the treatment of AD (35). In this study, our results showed that caspase-3 activity was significantly (p < 0.05) increased in brain tissue of Aβ peptide treated but not untreated rat group. 

ATP production via oxidative phosphorylation is the most important mitochondrial function. In addition, the ATP level determines the mode of cell death in target cells. In fact, ATP behaves as a switch between apoptosis and necrosis (36). We found that the ATP/ADP ratio decreased in isolated brain mitochondria in treated rat group.

## Conclusion

 In general, our findings showed that Aβ peptide treatment significantly increases brain mitochondrial ROS formation through disruption of MMP. This event leads to mitochondrial membrane depolarization, mitochondrial swelling, ATP/ADP decrease and expulsion of cytochrome c, and finally caspase-3 activation which can mediate apoptosis in brain neurons of rat. 
